# Aerial Imagery Reveals Abnormal Stingray, *Taeniura lymma* (Myliobatiformes: Dasyatidae), in the Central Red Sea

**DOI:** 10.1002/ece3.70411

**Published:** 2024-12-10

**Authors:** Ioana Andreea Ciocănaru, Brian Owain Nieuwenhuis, Raquel Lubambo Ostrovski, Jesse E. M. Cochran, Ashlie J. McIvor, Burton H. Jones

**Affiliations:** ^1^ Marine Science Program, Biological, Environmental Sciences and Engineering Division King Abdullah University of Science and Technology (KAUST) Thuwal Saudi Arabia; ^2^ Red Sea Research Center King Abdullah University of Science and Technology (KAUST) Thuwal Saudi Arabia; ^3^ Department of Environmental Protection and Regeneration Red Sea Global Tabuk Saudi Arabia

## Abstract

While morphological abnormalities have been widely reported in batomorphs, ontogenetic deformities of the posterior pectoral fin are rare. In this paper, we present a bluespotted ribbontail ray, *Taeniura lymma* (Forsskål, 1775), with symmetrically deformed posterior pectoral fins. The specimen was observed through aerial imagery on a coastal sandflat in the central Red Sea (22.30° N, 39.09° E). The bilateral symmetry of the deformity indicates it likely has a genetic base. However, lacking access to the specimen, the ultimate cause of the abnormality remains uncertain. The incomplete disk closure did not seem to affect survival, as the specimen reached a disk width of 22 cm, well above the regional size at sexual maturity. Our observations constitute both the first report of a morphological abnormality in *T. lymma* and the first record of a batomorph with a symmetrically deformed posterior pectoral fin. In this paper, we present a bluespotted ribbontail ray, *Taeniura lymma* (Forsskål, 1775), with symmetrically deformed posterior pectoral fins. The specimen was observed through aerial imagery on a coastal sandflat in the central Red Sea (22.30° N, 39.09° E). Our observations constitute both the first report of a morphological abnormality in *T. lymma* and the first record of a batomorph with a symmetrically deformed posterior pectoral fin.

## Graphical abstract

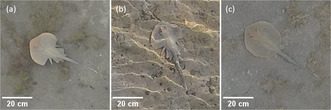

## 1 Introduction

Morphological defects in batomorphs have been reported for over 130 years (Bureau 1889), encompassing a wide range of deformities including misshapen genitalia, dental abnormalities, hermaphroditism, bicephaly, anophthalmia, albinism, and fin defects (Ehemann, García‐Rodríguez, and De La Cruz‐Agüero 2022; Ribeiro‐Prado et al. 2008). Reports of fin deformations include the absence of fins (Capapé et al. 2015; Ehemann, García‐Rodríguez, and De La Cruz‐Agüero 2022), the presence of aberrant fins (Ben Brahim and Capapé 1997; Deli Antoni et al. 2012), and deformed pectoral disks (Ramaiyan and Sivakumar 1988; Ribeiro‐Prado et al. 2008; Valderrama‐Herrera, Kanagusuku, and Ramírez‐Amaro 2022). Anterior incomplete disk closure, in which the pectoral fins failed to completely fuse with the head, is the most commonly reported defect (Ehemann, García‐Rodríguez, and De La Cruz‐Agüero 2022; Ribeiro‐Prado et al. 2008; Valderrama‐Herrera, Kanagusuku, and Ramírez‐Amaro 2022). Malformations along the posterior side of the pectoral disk are much rarer, but completely cleft posterior pectoral fins with a fringe‐like appearance have been reported in *Pateobatis jenkinsii* (Annandale, 1909) and *Dasyatis hypostigma* (Last et al. 2016) (Ramaiyan and Sivakumar 1988; Ribeiro‐Prado et al. 2008).

The bluespotted ribbontail ray *Taeniura lymma* (Forsskål, 1775) is a small, benthic batomorph that is easily recognizable by its greenish‐brown coloration punctuated with bright blue spots and tail stripes (Surapaneni et al. 2024). It is broadly distributed throughout the Indo‐Pacific and is generally associated with shallow coral reefs and adjacent habitats (Dabruzzi et al. 2013; Last et al. 2016). Juveniles tend to nursery in extremely shallow waters, including intertidal zones and other nearshore habitats (Dabruzzi et al. 2013; Leurs et al. 2023; O'Shea et al. 2012). Despite being common and broadly distributed in easily accessible habitats, *T. lymma* is poorly studied. This is likely due to the lack of conservation concern (Sherman et al. 2021). Here, we report the first observations of incomplete disk closure in an individual of this species and the first instance of a symmetrically deformed posterior pectoral disk in any batomorph.

## 2 Methods

### 2.1 Study Area

**FIGURE 1:**
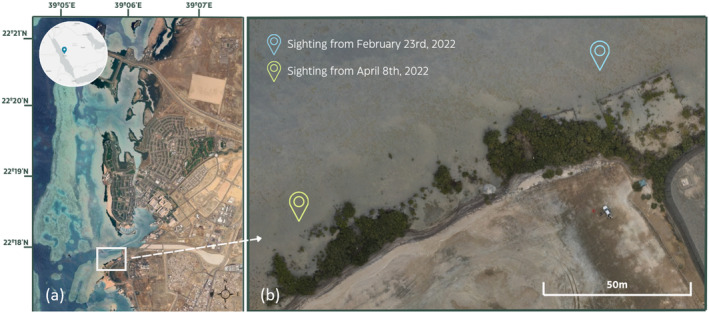
(a) ESRI World Imagery showing KAUST campus and surrounding reefs. White square indicates the study area, inset shows general location in the central Red Sea. (b) UAV orthomosaic of the study area showing the locations where the deformed ray was sighted.

An abnormal bluespotted ribbontail ray was encountered by chance while inspecting unoccupied aerial vehicle (UAV) imagery collected to monitor mangroves. All UAV surveys were conducted over a small *Avicennia marina* (Forssk.) Vierh. mangrove stand (~12,000 m^2^) within the campus of the King Abdullah University of Science and Technology (KAUST) on the Saudi Arabian coast of the central Red Sea (22.30° N, 39.09° E) (Figure 1). While centered on the mangrove fringe the UAV surveys included a part of the adjacent sandflat, where the bluespotted ribbontail ray was spotted. The depth of this sandflat ranges from 10 to 40 cm, and it is bordered by a deeper shipping channel (Figure 1a). Nearby sandflats (< 2 km) were previously shown to harbor four batomorph species, with *T. lymma* being the most common (McIvor et al. 2022).

### 2.2 UAV Surveys

Four aerial surveys were conducted, on February 8, February 23, April 8, and December 15, 2022. UAV surveys were always conducted in the morning between 8 and 10 a.m. to minimize sun glint (Joyce et al. 2019). We used a DJI Matrice 300 RTK equipped with a 45‐megapixel, full‐frame DJI Zenmuse P1 camera with a DJI DL 35 mm F2.8 LS ASPH lens (DJI 2021). The surveys were designed in the DJI Pilot 2 application and consisted of a lawn mower pattern at 20‐m altitude above ground/water level (AGL) with 80% frontal overlap and 75% side overlap. The horizontal speed of the UAV was 5.5 m/s, and each survey took approximately 15 min to complete.

### 2.3 Ray Detection and Measurements

The 4110 images captured over the four flights were visually inspected for the presence of rays in the order of their acquisition. The high spatial resolution of our camera, the low flight altitude, and shallow study area allowed easy detection of rays. Additionally, the images from each flight were processed in Agisoft Metashape Professional to generate orthomosaics (Agisoft 2023; Casella et al. 2017). The orthomosaics were then used to plot the location of all ray detections. This allowed us to filter out duplicate detections of the same individual in parallel UAV transects. To measure each ray, we selected the image where the ray was close to the center of the frame (to minimize optical distortion caused by refraction) and its body or tail was not folded (for accuracy of measurement). The best image was loaded in the software FIJI, where disk width (DW) and total length (TL) were measured (Schindelin et al. 2012). The obtained pixel values were converted to standard linear units by multiplication with the Ground Sampling Distance, which was 0.25 cm/pixel (Equation 1) (DJI 2021).

(1) $$\mathrm{GSD}=\frac{\text{Sensor width}\;\left(\mathrm{mm}\right)\times \text{Flight altitude}\;\left(m\;\mathrm{AGL}\right)}{\text{Focal length}\;\left(\mathrm{mm}\right)\times \text{Image width}\;\left(\text{pixels}\right)}$$

## 3 Results

**FIGURE 2:**
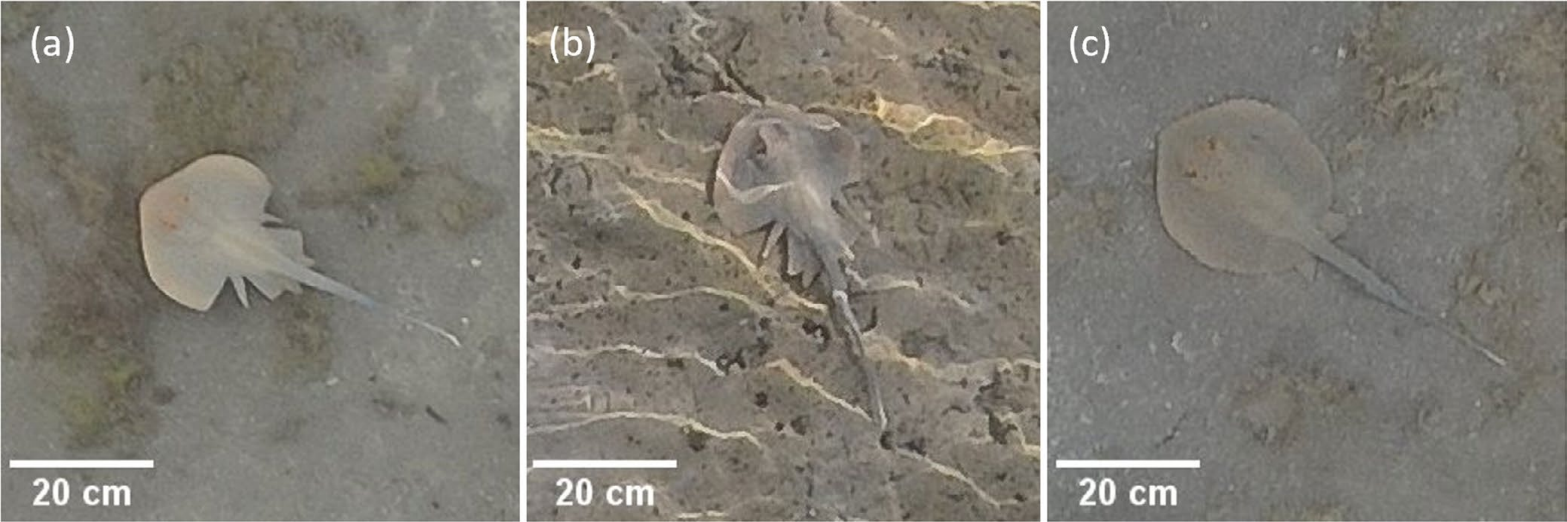
UAV images of the *T. lymma* specimen seen during our surveys. (a) The deformed specimen seen on February 23, 2022. (b) The deformed specimen seen on April 8, 2022. (c) A typical specimen seen on February 23, 2022, as reference.

On February 23, 2022, we observed one *Taeniura lymma* (Forsskål, 1775) individual with symmetrically deformed pectoral fins (Figures 1b and 2a). The deformed *T. lymma* exhibits two major pectoral fin abnormalities. The first is a noticeable split at the midpoint of the pectoral fin, creating a deep inset that significantly alters the smooth, rounded contour typical of this species. A second split is present toward the posterior end of the fin, creating a second indentation that further disrupts the fin's shape. Between these two indentations lies a small lobe‐like section, which appears distinctly smaller than the rest of the fin structure and gives the appearance of an extra, separated segment of the pectoral fin.

Despite the significant deformities, the trailing edges of the bifurcated pectoral fin are still present and positioned closer to the midline posteriorly, maintaining consistency with the anatomy of a typical individual. Although no true claspers are visible in the aerial imagery, it cannot be entirely discounted that they may be tucked beneath the ray's body or tail, obscured from view.

**FIGURE 3:**
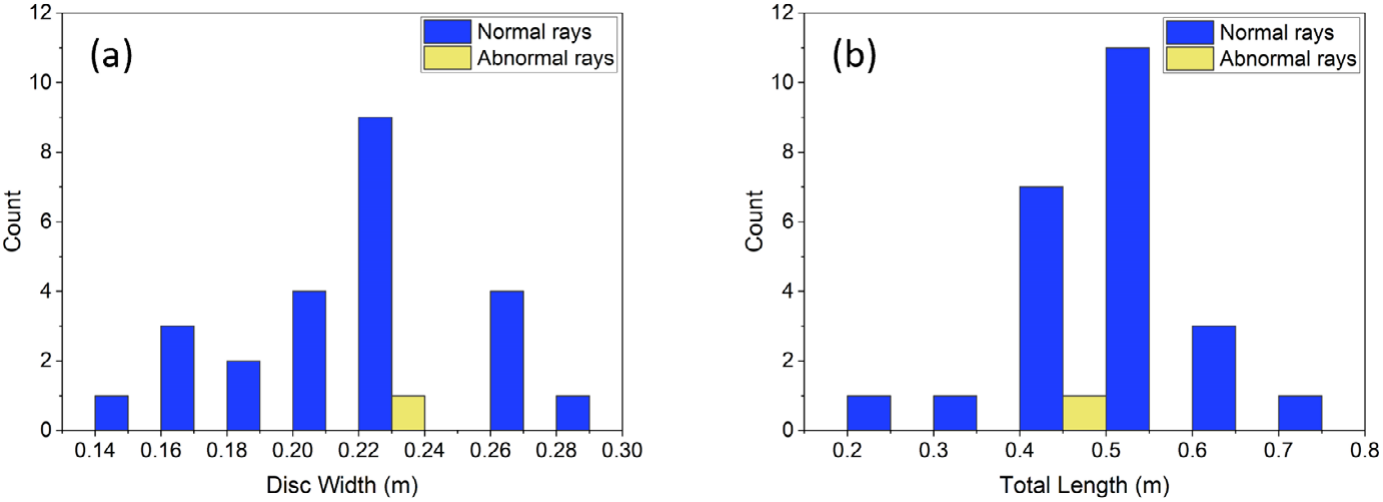
(a) Disk width and (b) total length of the deformed and normal rays observed during the UAV surveys.

The deformed specimen had a disk width of 22 cm with total length of 48.5 cm. The size of the deformed ray falls neatly within the size range of the non‐deformed *T. lymma* specimens we observed in the area which had an average disk width of 21.7 cm (SD = 3.5, *n* = 24) and an average total length of 51.6 cm (SD = 11.3, *n* = 24) (Figure 3).

**FIGURE A1:**
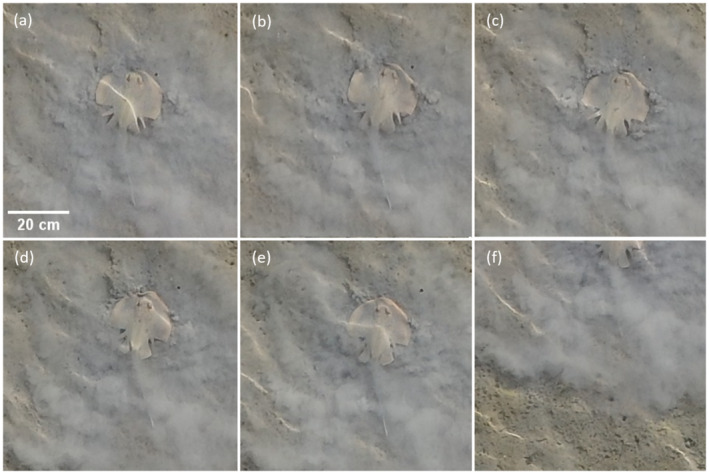
(a–f) Consecutive frames showing the deformed *T. lymma* seen during the drone survey of April 8, 2022. (c, d) capture the uplifted snout, referred to as “balancing behavior,” associated with feeding (Madduppa et al. 2020).

On April 8, 2022, another observation of a deformed bluespotted ribbontail ray was made (Figures 1b and 2b). Based on morphological resemblance and a similar size (DW = 22 cm, TL = 48 cm), we deem it most likely this observation was a “recapture” of the first specimen instead of a second deformed individual. During the survey of April 8, the deformed ray was observed digging into the sediment with its snout uplifted, a balancing behavior associated with feeding in *T. lymma* (Figure A1 in Appendix) (Madduppa et al. 2020).

## 4 Discussion

Here, we provide the first ever evidence for an ontogenetic deformity in the bluespotted ribbontail ray, *T. lymma*. We describe a specimen with a symmetrical deformity of the posterior pectoral disk. The individual had grown to a disk width of 22 cm, which is well above the 14.3 ± 2.8 cm size at birth reported for bluespotted ribbontail rays (Santos Ferreira and Cabral 2013) and exceeds the regional size at sexual maturity of 20.3 cm (McIvor et al. 2023). Thus, the abnormal pectoral disk did not seem to affect its survival, despite the pivotal role of the (posterior) pectoral fin in batomorph feeding and locomotion (Blevins and Lauder 2012; Freitas et al. 2019; Rosenberger and Westneat 1999). This may be because, in contrast to most deformities (Ribeiro‐Prado et al. 2008), the resulting body shape of our specimen does not appear to be hydrodynamically inefficient but instead somewhat resembles the body plan of certain skate species (Last et al. 2016).

Contact with anthropogenic pollutants, poor nutrition, genetic disorders, diseases, and stunted embryonic development have all been reported as potential causes of morphological abnormalities in elasmobranchs. However, data to corroborate these hypotheses are usually lacking, so the ultimate cause of batomorph deformities often remains uncertain (Ehemann, García‐Rodríguez, and De La Cruz‐Agüero 2022). The same is true for this study, as we did not have access to the specimen to obtain samples for molecular, anatomical, or physiological analysis. However, the almost perfect bilateral symmetry of the deformity seems to indicate this abnormality has a genetic basis. Chemical pollution is unlikely to be the main driver behind this deformity. Our study area is located between Rabigh (~60 km) and Jeddah (~100 km) where marine sediments were previously classified as highly polluted (Youssef and El‐Sorogy 2016). Coastal sediments closer to our study area (< 15 km) were shown to have only low‐to‐moderate levels of contamination (Ruiz‐Compean et al. 2017).

Only two other cases of deformed posterior pectoral fins have previously been reported for batomorphs (Ramaiyan and Sivakumar 1988; Ribeiro‐Prado et al. 2008). In both cases, the posterior pectoral fin was completely cleft giving a fringe‐like appearance, which starkly contrasts the symmetrical deformity presented here. Perhaps, the two most similar deformities previously reported are a butterfly ray, *Gymnura poecilura* (Shaw, 1804), and a Chilean eagle ray, *Myliobatis chilensis* Philippi, 1892, whose pectoral fin was symmetrically split in the middle (Behera et al. 2024; Valderrama‐Herrera, Kanagusuku, and Ramírez‐Amaro 2022). Somewhat similar are the most severe, symmetrical cases of an incomplete fusion of the pectoral fin to the head, which is relatively common in batomorphs (Bureau 1889; Clarke 2021; Ehemann, García‐Rodríguez, and De La Cruz‐Agüero 2022; Legendre 1935; Ribeiro‐Prado et al. 2008; Templeman 1965; Valderrama‐Herrera, Kanagusuku, and Ramírez‐Amaro 2022). The prevalence of incomplete disk closures in batomorphs is likely a result of the fact that the pectoral disk develops out of two separate pectoral fins that first grow in length and width and then gradually fuse with the body (Thorson, Langhammer, and Oetinger 1983). However, the development of the anterior and posterior pectoral fin is underpinned by different genetic pathways (Nakamura et al. 2015). The anterior growth of the pectoral fin is controlled by a unique molecular pathway driven by expression of Fgf7 and 3′Hox genes. In contrast, the posterior region develops under the influence of Gli3, Shh, and 5′Hox genes, which are part of a highly conserved molecular pathway that is similar to the genetic underpinnings of tetrapod limb development (Nakamura et al. 2015). This may explain why ontogenetic deformities of the posterior pectoral fin, such as we describe here, are so much rarer than deformities of the anterior fin.

Finally, our study highlights the multi‐modality of UAV surveys. By providing a permanent record of the environment UAV imagery enabled us to spot this uniquely deformed ray that may otherwise have gone unnoticed, even though it inhabited a university campus.

## Author Contributions

**Ioana Andreea Ciocănaru:** investigation (lead), writing – original draft (equal), writing – review and editing (equal). **Brian Owain Nieuwenhuis:** investigation (supporting), writing – original draft (equal), writing – review and editing (equal). **Raquel Lubambo Ostrovski:** writing – original draft (equal), writing – review and editing (equal). **Jesse E. M. Cochran:** validation (equal), writing – review and editing (equal). **Ashlie J. McIvor:** validation (equal), writing – review and editing (equal). **Burton H. Jones:** funding acquisition (lead), supervision (lead), writing – review and editing (equal).

## Conflicts of Interest

The authors declare no conflicts of interest.

## Data Availability

All data is included in the paper's main text and appendices.
